# The Contribution of Diet and Genotype to Iron Status in Women: A Classical Twin Study

**DOI:** 10.1371/journal.pone.0083047

**Published:** 2013-12-31

**Authors:** Susan J. Fairweather-Tait, Geoffrey R. Guile, Ana M. Valdes, Anna A. Wawer, Rachel Hurst, Jane Skinner, Alexander J. Macgregor

**Affiliations:** 1 Norwich Medical School, University of East Anglia, Norwich, United Kingdom; 2 Department of Twin Research & Genetic Epidemiology, King's College, London, United Kingdom; Instituto de Higiene e Medicina Tropical, Portugal

## Abstract

This is the first published report examining the combined effect of diet and genotype on body iron content using a classical twin study design. The aim of this study was to determine the relative contribution of genetic and environmental factors in determining iron status. The population was comprised of 200 BMI- and age-matched pairs of MZ and DZ healthy twins, characterised for habitual diet and 15 iron-related candidate genetic markers. Variance components analysis demonstrated that the heritability of serum ferritin (SF) and soluble transferrin receptor was 44% and 54% respectively. Measured single nucleotide polymorphisms explained 5% and selected dietary factors 6% of the variance in iron status; there was a negative association between calcium intake and body iron (p = 0.02) and SF (p = 0.04).

## Introduction

Levels of iron in the body are tightly regulated through changes in the efficiency of absorption to ensure sufficient iron is available for vital functions but at the same time to avoid the accumulation of excessive iron. It is well established that genetics plays a significant role in iron overload, the most common example being hereditary hemochromatosis which, in spite of its low penetrance, is attributable to mutations in the hemochromatosis gene, *HFE* [1], but the detailed role of genetics in determining body iron status remains largely unexplained. Iron absorption is also dependent on dietary composition as a number of food constituents render luminal iron more or less available for duodenal uptake by the iron transport protein, Divalent metal transporter 1 (DMT1) [2]. Estimates of the relative contribution of diet and genotype to iron status vary widely according to data from different studies, but they have not yet been studied simultaneously in the same population group. The aim of the present study was to assess the role of dietary and genetic factors in determining iron levels using a classical twin study design, and to quantify the relative contribution of each by including specific nutritional factors and candidate genes.

## Materials and Methods

A sample of 100 MZ and 100 DZ female healthy twin pairs was selected from a group of around 3,500 twins enlisted in the TwinsUK registry. The key inclusion criteria were data on body mass index (BMI), dietary intake and genotype, together with a serum blood sample. The sample size was chosen to provide power of 90% and type-I error rate of 0.05 [3] in detecting a heritability of approximately 30%. The sample was selected at random but weighted in favour of premenopausal twins as their iron requirements are higher and hence they are more sensitive to environmental modulators. MZ and DZ pairs were also age- and BMI-matched in the selection process to achieve balance between the zygosity groups. All participants gave written, informed consent, and the Guy's and St Thomas' Hospital ethics committee approved the study (REC ref. EC04/015).

Dietary information was collected from a validated 131-item food frequency questionnaire (FFQ), previously developed for the European Prospective Investigation into Cancer and Nutrition Study [4], from which nutrient intakes were determined using an established database [5]. Individuals completed questionnaires that included information on menopausal history. Intakes of dietary constituents that might influence iron status, by modifying the quantity of iron absorbed, were calculated: iron, calcium, dietary fibre (non-starch polysaccharide), vitamin C, and frequency of red meat (beef, pork, lamb) intake.

Blood samples were collected at approximately the same time as the FFQ and other questionnaires and stored at −80°C. As part of other on-going research, some of the samples had been analysed for C-reactive protein, (data shown in Table 1). Analysis of serum ferritin (SF) and soluble transferrin receptor (sTfR) were performed using enzyme immunoassays (Spectro ferritin and TfR respectively, Ramco USA). Two additional standards (10 ng and 100 ng) were included in addition to the standards provided in the SF kit to increase the assay accuracy. A WHO reference reagent (recombinant soluble transferrin receptor, NIBSC code: 07/202) was run in duplicate on every sTfR plate at 30 µg and 90 µg/L to standardise the immunoassay (average CV 5.7%). The sTfR value was calculated from the WHO conversion [6]. Duplicates were analysed in 30% of SF samples (average CV 6.5%) and in 20% of sTfR samples (average CV 5.2%). Body iron, widely accepted as a good measure of iron status, was calculated from the sTfR/log SF index [7].

**Table 1:** Characteristics of the twins.

	All	MZ twins	DZ twins
No. of individuals	400	200	200
Post-menopausal (%)	64 (16)	32 (16)	32 (16)
Age (y)	47.8±9.3	47.6±9.6	48.1±9.1
	(28.6, 72.8)	(28.6, 72.6)	(33.5, 72.8)
BMI (kg/m^2^)	24.9±24.8	24.8±4.6	24.9±5.2
	(16.5, 44.4)	(17, 44.4)	(16.5, 43.5)
Smoking status:			
Never (%)	249 (62.3)	136 (68.0)	113 (56.5)
Former (%)	107 (26.8)	45 (22.5)	62 (31.0)
Current (%)	44 (11.0)	19 (9.5)	25 (12.5)
C-reactive protein (mg/l)	2.83±4.37	2.34±2.93	3.31±5.39
(n = 136 MZ, 140 DZ)	(0.16, 34)	(0.16, 20.9)	(0.16, 34.0)
Dietary calcium	1074±328	1070±324	1078±333
(mg/day)	(232, 2119)	(232, 2010)	(232, 2119)
Dietary fibre (g/day)	19.1±7.1	19.3±6.9	18.9±7.2
	(5.3, 51.0)	(5.3, 51.0)	(5.4, 50.5)
Dietary vitamin C	154.9±77.9	160.4±77.1	149.4±78.5
(mg/day)	(31.0, 561)	(41.2, 515)	(31.0, 561)
Meat consumption	2.0±1.72	1.9±1.5	2.2±1.9
(times/week)	(0, 11.5)	(0, 8.75)	(0, 11.5)

Values are means ± SD (min, max).

Single nucleotide polymorphisms (SNPs) selected for this analysis were from genes known to be involved in iron metabolism (http://www.micronutrientgenomics.org/index.php/Iron). The list was filtered to include only SNPs with a minor allele frequency (MAF) >5%. The pairwise linkage disequilibrium was examined and where r^2^>0.2, the SNP with the greater MAF was retained. A total of 15 iron- related SNPs were included in the analysis.

The twin model partitions variance in a trait into genetic and environmental components. The contribution of genetic and environmental factors was assessed through variance component analysis implemented in SOLAR v4 [8]. The quantitative age- and BMI-adjusted phenotype (y) was modelled as a linear function of a polygenetic genetic effect (g) and a random environmental component (e) (Model 1). This was extended to examine the influence of 5 individual nutrients (Model 2), and the 15 candidate SNPs (Model 3)

Model 1: 
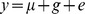


Model 2: 
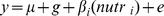


Model 3: 
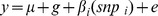


SOLAR models the covariance among phenotype values (Ω) using the kinship coefficient matrix (Φ) (derived from the family structure, Ω = 2Φ*σ_g_^2^*+*I σ_e_^2^*), where *σ_g_^2^* and *σ_e_^2^* are the variances of the polygenic and environmental effects and *I* is the identity matrix. The trait heritability was estimated from *σ_g_^2^*/(*σ_g_^2^*+*σ_e_^2^*) in Model 1. The significance of individual covariates was assessed by examining the change in model likelihood after the covariates were omitted.

Measures of genotypic association were tested under an additive model.

To account for multiple testing, the Benjamini-Hochberg adjustment was applied to p values using the p.adjust function in R, assuming a 10% false discovery rate. The proportional contribution of measured nutritional factors and measured SNPs to overall variance was estimated from the variance of *β_i_* (*nutr_i_*) and *β_i_* (*snp_i_*) in Models 2 and 3 respectively.

## Results and Discussion

Phenotypic data for the twins and the SF and TfR concentrations are shown in Table 1. There were no significant differences between MZ and DZ twins for any of the measured variables, including factors that might affect iron metabolism such as smoking status (assessed in all individuals) and C-reactive protein, a measure of inflammatory status (measured in some of the twins). Parity was similar in MZ and DZ twins, with an average of 1.6 pregnancies. Iron supplements were reported to have been used by only 5 MZ and 4 DZ individual twins, all of whom were premenopausal, and had SF concentrations that were below the overall mean (mean 39.2 µg/L). These individuals were retained in the analysis. SF concentrations <12 µg/L (depleted iron stores) were present in 18% of the total sample; the proportion was similar in MZ and DZ groups. However, none of the twins had been clinically diagnosed with iron deficiency anaemia.

The heritability of iron status (Model 1) was calculated to be 46 (95% CI 28, 65)% for SF, 54 (95% CI 40, 67)% for sTfR, and 44 (95% CI 26, 61)% for body iron.

The overall contribution of the selected dietary factors to variance in iron status was 6% (Model 2). There was a negative association between calcium intake and body iron (p = 0.004) and SF (p = 0.008) which remained significant (p = 0.02 and 0.04 respectively) after taking multiple testing into account.

The contribution of the 15 SNPs examined to variance in iron status was 5% (Model 3). Three were significant for one or more measures of iron status (rs960748 *CYBRD1*, rs9366637 *HFE*, rs4434553 *TFR2*) (Table 2) although none remained significant after taking into account multiple testing.

**Table 2:** Contribution from SNPs to body iron, serum ferritin (SF) and soluble transferrin receptor (sTfR): *unadjusted p values.

SNP	Gene	Gene name	Body iron	SF	sTfR
rs960748	*CYBRD1*	cytochrome b reductase 1	0.008	0.079	0.168
rs2304704	*SLC40A1*	solute carrier family 40 (iron-regulated transporter)	0.783	0.566	0.623
rs8177190	*TF*	transferrin	0.565	0.558	0.294
rs1799852	*TF*	transferrin	0.420	0.174	0.228
rs3811647	*TF*	transferrin	0.990	0.753	0.237
rs1049296	*TF*	transferrin	0.805	0.796	0.466
rs1115219	*TF*	transferrin	0.367	0.948	0.424
rs3817672	*TFRC*	transferrin receptor (p90, CD71)	0.892	0.949	0.105
rs9366637	*HFE*	human hemochromatosis protein	0.063	0.028	0.516
rs1572982	*HFE*	human hemochromatosis protein	0.281	0.167	0.115
rs4727457	*TFR2*	transferrin receptor protein 2	0.627	0.191	0.863
rs4434553	*TFR2*	transferrin receptor protein 2	0.465	0.945	0.034
rs829021	*SLC11A2*	solute carrier family 11 (proton-coupled divalent metal ion transporters), member 2	0.093	0.613	0.154
rs149411	*SLC11A2*	solute carrier family 11 (proton-coupled divalent metal ion transporters), member 2	0.718	0.686	0.976
rs7251432	*HAMP*	hepcidin antimicrobial peptide	0.507	0.927	0.174

Adjusted p values were all >0.05.

This is the first published report examining the combined effect of diet and genotype on body iron content using a classical twin study design. The results demonstrate that there is a substantial genetic contribution to iron status, which agrees with Whitfield *et al* [9] who reported that 47% of the variance in SF in twins was explained by additive genetic factors. However, in their study sTfR was not measured, therefore body iron could not be calculated, nor was there any assessment of the dietary impact on iron stores.

Our study provides evidence to support a contribution for genes known to be involved in iron metabolism. In particular, three (rs960748 *CYBRD1*, rs9366637 *HFE*, rs4434553 *TFR2*) are worthy of further investigation. These genes have also emerged in other studies. In a multi-ethnic population of iron-deficient and normal iron status adults SNPs in the *TR* and *HFE* genes were identified that were associated with at least one iron phenotypic measure [10]. In a Genome Wide Association Study (GWAS) in MZ twins and their siblings 40% of the genetic variation in serum transferrin concentration was explained by a combination of three SNPs in the transferrin (*TF*) gene and the *HFE* C282Y mutation, but the proportion of phenotypic variance in SF and other measures of iron status could not be explained by SNPs other than C282Y [11]. In a meta-analysis of 5 GWAS Oexle *et al* [12] found that sTfR concentration was associated with *HFE* and *TMPRSS6* genes and the *PCSK7* locus. The involvement of these genes suggests that they might have a role in screening for susceptibility to iron deficiency. However, a substantial proportion of genetic variation remains unexplained, justifying the pursuit of more detailed genetic analysis in large scale genome wide studies.

Among the potential environmental factors influencing iron status, this study identified calcium as being negatively associated with iron status, which agrees with its reported inhibitory effect on iron absorption in single meal studies, for example, Minihane & Fairweather-Tait [13]. Data from calcium supplementation studies are inconsistent [14], and there are conflicting reports about the threshold dose of calcium that affects absorption from a 5 mg dose of iron, ranging from as low as 165 mg [15] up to 800 mg [16]. One of the proposed mechanisms of action for the acute effect of calcium is that it internalises DMT1, thereby limiting iron transport across the apical membrane of the mucosal cell [17]. It could be argued that the consequence of this would be a compensatory increase in DMT1 expression when the cells are next exposed to digestive chyme, but the degree to which this is achievable is dependent on the dietary supply of available iron. It was notable that the selected dietary factors accounted for only 6% of the overall variation in iron status in this group. This may in part be a reflection of the relatively narrow range of dietary intakes in this group of healthy female twins. Nevertheless, as they are typical of the adult UK population, our findings suggest that modifications in a Western diet are not required to maintain iron status, with the possible exception of calcium intakes in individuals at high risk of iron deficiency, although it is possible that calcium intake in the twins was a proxy measure of differences in dietary patterns and/or intakes of other dietary modulators of iron absorption.

Whitfield *et al* [18] reported that the combined effect of age, menopausal status and magnitude of blood loss accounted for up to 18% of the variance in SF, and Harvey *et al* [19] found that menstrual loss accounted for 11.5% in the variance in SF. They reported that diet was responsible for a further 6.7%, a figure that agrees well with our estimate of 6%. Our analysis took age into account but we could not examine the effect of menstrual blood loss, although this is known to have a high degree of heritability [20]. It is also conceivable that interactions between diet and genes might account for part of the unexplained variation. However, gene environment interactions cannot be addressed in these data and would require a substantially larger sample size.

The results of our study quantify heritability of iron status using the most appropriate measures of body iron. With the classical twin study design we have been able to gain a unique insight into the effect of environmental factors and genotype on iron status, and demonstrated that they make an approximately equal contribution.
